# Association of Low Bone Mass with Decreased Skeletal Muscle Mass: A Cross-Sectional Study of Community-Dwelling Older Women

**DOI:** 10.3390/healthcare8030343

**Published:** 2020-09-16

**Authors:** Koji Nonaka, Shin Murata, Hideki Nakano, Kunihiko Anami, Kayoko Shiraiwa, Teppei Abiko, Akio Goda, Hiroaki Iwase, Jun Horie

**Affiliations:** 1 Department of Rehabilitation, Faculty of Health Sciences, Naragakuen University, Nara 631-8524, Japan; anami@naragakuen-u.jp; 2 Department of Physical Therapy, Faculty of Health Sciences, Kyoto Tachibana University, Kyoto 607-8175, Japan; murata-s@tachibana-u.ac.jp (S.M.); nakano-h@tachibana-u.ac.jp (H.N.); shiraiwa@tachibana-u.ac.jp (K.S.); abiko@tachibana-u.ac.jp (T.A.); goda@tachibana-u.ac.jp (A.G.); horie-j@tachibana-u.ac.jp (J.H.); 3 Department of Physical Therapy, Faculty of Rehabilitation, Kobe International University, Kobe 658-0032, Japan; iwase@kobe-kiu.ac.jp

**Keywords:** older women, bone mass, skeletal muscle index, grip strength, knee extension strength, gait speed, timed up and go

## Abstract

This study aimed to investigate the characteristics of skeletal muscle mass, muscle strength, and physical performance among community-dwelling older women. Data were collected from 306 older adults, and the data of 214 older women were included in the final analysis. Participants’ calcaneus bone mass was measured using ultrasonography. Based on their T-scores, participants were divided into the following three groups: normal (T-score > −1), low (−2.5 < T-score ≤ −1), and very low (T-score ≤ −2.5) bone mass. Further, participants’ skeletal muscle mass, muscle strength (grip and knee extension strength), and physical performance [gait speed and timed up and go (TUG)] were measured. Arm skeletal muscle index (SMI, skeletal muscle mass/height^2^), leg SMI, and appendicular SMI in the very low bone mass group were low compared to those of the low bone mass group (*p* = 0.034, *p* = 0.011, and *p* = 0.009, respectively). Grip and knee extension strength, gait speed, and TUG were not significantly different between the groups. These findings suggest that older women with low bone density had decreased skeletal muscle mass. Therefore, maintaining or improving skeletal muscle mass may prevent low bone mass.

## 1. Introduction

Loss of bone tissue and bone strength occurs with aging, and is accelerated in menopausal women [1]. Osteoporosis, a condition marked by low bone mass, is three times more common in women compared to in men [2]. Low bone density can result in hip fractures [3]. A study reported that among adults who could previously perform activities of daily living (ADL) independently, only 36% returned to independence, 27% survived but required ADL assistance, and 37% died 6 months to 2.5 years following a hip fracture [4]. Therefore, it is important for older women to maintain bone health to prevent fractures.

Muscle mass also reduces with aging [5,6], and age-related loss of bone and muscle mass occurs almost simultaneously [7]. Muscle mass is related to bone mass in postmenopausal women [8]. However, studies have reported inconsistent findings on the association between muscle mass and bone mineral density in older women. One study reported that muscle mass is related to bone mineral density in older adults [9], whereas another study reported that age-related muscle loss does not seem to be involved in bone mass loss [10].

It is helpful for health professionals to understand the characteristics of muscle mass in older women diagnosed with low muscle mass, in order to maintain their bone health and reduce the risk of fractures. The aim of this study was to investigate the characteristics of muscle mass in older women who have low bone mass. Furthermore, we investigated the characteristics of muscle strength and physical performance in these women, because it is possible that muscle mass is related to muscle strength, which influences physical performance [11].

## 2. Materials and Methods

### 2.1. Participants

**Figure 1:**
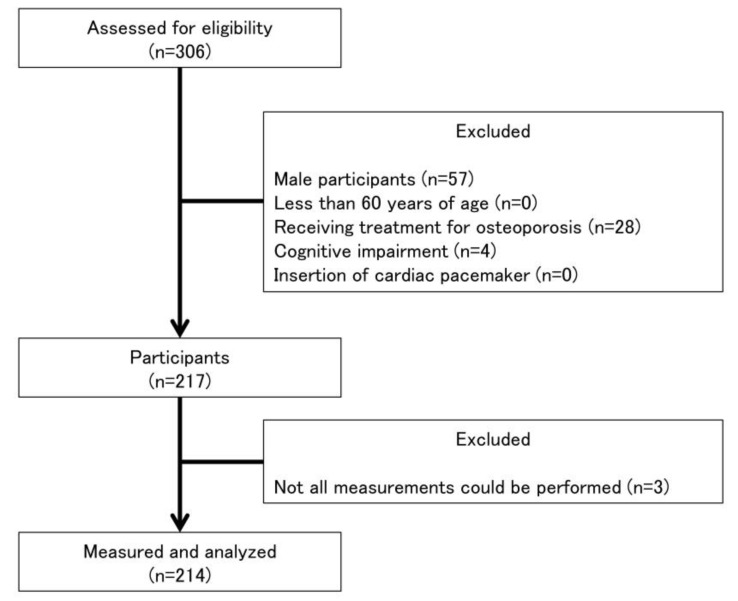
Flowchart of participation criteria.

The design of this study was cross-sectional and was conducted in 2019 in accordance with the Declaration of Helsinki and was approved by the ethics committee of the Kyoto Tachibana University (approval number: 18–26). Public information papers were used to recruit 306 community-dwelling older adults. Data on participants’ age, height, and weight were collected after which participants completed the Mini-Mental State Examination (MMSE). In addition, information regarding the prevalence of disease, such as osteoporosis, hypertension, hyperglycemia, orthopedic disease, diabetes mellitus, cardiovascular disease, pulmonary disease renal disease, and cancer, was obtained from the participants. Inclusion criteria were as follows: (1) age ≥ 60 years, (2) no cognitive impairment (MMSE score ≥ 24), and (3) ability to complete all measurements. The exclusion criteria were: (1) male sex, (2) implanted with a cardiac pacemaker, and (3) receiving treatment for osteoporosis. The final analysis used data from 214 participants (Figure 1).

### 2.2. Bone Mass

Bone mass was measured at the right calcaneus by bone quantitative ultrasonometry using Benus evo (Nihon Kohden Co., Tokyo, Japan). T-scores, which is the standard deviation when compared with the young adult mean, were obtained, based on which participants were divided into three groups in accordance with the criteria published by the World Health Organization (WHO): normal (T-score > −1), low (−2.5 < T-score ≤ −1), and very low (T-score ≤ −2.5) bone mass [2]. The diagnostic criteria of T-scores published by the WHO have not been recommended to apply to T-scores obtained from quantitative ultrasound [12]. However, we used the WHO’s criteria as cut-offs to divide participants into groups, because the cut-offs of parameters obtained from quantitative ultrasound are unclear.

### 2.3. Skeletal Muscle Mass

Skeletal muscle mass was determined using InBody 470 (InBody Japan Inc., Tokyo, Japan) as described in a previous study [13]. Skeletal muscle mass index (SMI) values were calculated by dividing skeletal muscle weight by the square of height (kg/m^2^).

### 2.4. Muscle Strength

Grip strength and knee extension strength were used to determine participants’ physical performance, as described in Abe et al. [14]. Participants gripped a hand grip dynamometer (T.K.K.58401, Takei Scientific Instruments Co., Ltd., Niigata, Japan) as hard as possible and grip strength were recorded. Grip strength of both right and left limbs were measured, and the highest value was used for the analysis.

Knee extension strength was measured using a hand-held dynamometer (μTas F-1; Anima Corp., Tokyo, Japan) as described in Bohannon [15]. Participants sat with their knees and hips flexed at 90° and participants performed maximal isometric muscle contraction. Measurements were performed for both right and left legs, and the highest value was used for the analysis.

### 2.5. Physical Performance

Gait speed and the timed up and go (TUG) test were used to determine participants’ physical performance. Participants walked at their usual speed and as fast as possible along an 11 m course, which included 3 m of acceleration and 3 m of deceleration zones at each end. The walking time spent in the middle 5 m course was measured using a digital stopwatch. Gait speed was expressed as m/s.

The TUG test was performed with a minor modification [16]. In a previous study, participants performed at the usual pace [16], whereas they were asked to perform at the fastest pace in the present study. Participants stood up from a chair without arm rests, walked along a 3 m course, turned around, walked back, and sat on the chair again as fast as possible. The time taken to complete the TUG test was measured using a digital stopwatch.

### 2.6. Sarcopenia

Sarcopenia was evaluated according to criteria published by the Asian Working Group for Sarcopenia in 2014 [17]. Participants were evaluated as having sarcopenia when they had low muscle mass (appendicular SMI < 5.7 kg/m^2^) with either low muscle strength (grip strength < 18 kg) or low physical performance (usual gait speed < 0.8 m/s) [17].

### 2.7. Statistical Analysis

One-way analysis of variance (ANOVA) was performed to compare age, height, weight, and body mass index (BMI) among the groups. If ANOVA found significant differences, a Bonferroni multiple comparison test was performed to determine significant differences between the three groups. A chi-squared test was performed to compare the prevalence of diseases and percentage of participants who met the criteria of sarcopenia between the groups. Analysis of covariance (ANCOVA) was performed to compare arm SMI, leg SMI, appendicular SMI, grip strength, knee extension strength, usual and fastest gait speed, and TUG duration between the three groups, adjusted for age, because it was presumed that measurement of bone mass by ultrasonography is affected by age [18]. If ANOVA found significant differences, a Bonferroni multiple comparison test was performed to determine significant differences between the three groups. SPSS ver. 25.0 (IBM Japan, Ltd., Tokyo, Japan) was used for statistical analysis, and *p* < 0.05 was set as statistical significance.

## 3. Results

Participants’ characteristics are presented in Table 1. Age and height were not significantly different between the three groups. Weight and BMI were significantly lower in the very low bone mass group than the low bone mass group (*p* < 0.001 and *p* = 0.001, respectively). The prevalence of diseases was not significantly different between the three groups. In the low bone mass group, one of the participants with orthopedic disease had a fracture.

A comparison of SMI, muscle strength, and physical performance between the normal, low, and very low bone mass groups is shown in Table 2. Arm SMI, leg SMI, and appendicular SMI were significantly lower in the very low bone mass group compared to those of the low bone mass group (*p* = 0.034, *p* = 0.011, and *p* = 0.009, respectively). These findings suggest that muscle mass decreases when bone mass decreases. Meanwhile, grip strength and knee extension strength were not significantly different between the three groups. In addition, usual and fastest gait speed and TUG were not significant different between the groups. These findings indicate that it is possible that physical performance does not decrease when bone mass decreases. The rates of sarcopenia were 0, 5.4, and 3.6% in the normal, low bone mass, and very low bone mass groups, respectively. The rates of sarcopenia were not significantly different between the three groups.

## 4. Discussion

The aim of this study was to compare the characteristics of SMI, muscle strength, and physical performance among older women with normal, low, and very low bone mass. There were several major findings in this study. First, SMI was low in the very low bone mass group compared to the low bone mass group. Second, muscle strength was similar for all groups. Finally, physical performance of all groups was similar. The arm, leg, and appendicular SMI were significantly lower in the very low bone mass group compared to those in the low bone mass group. Taniguchi et al. [19] reported that participants with osteoporosis had lower appendicular SMI compared to those without osteoporosis. In addition, some studies reported that bone mineral density was correlated with SMI [20] and lean muscle mass [21,22]. Our results support the findings of these studies and suggest that older women with low bone mass have low muscle mass. A study by Kaji [23] reported that age-related loss of muscle mass appears to occur earlier than bone mass loss due to aging. Taken together, decreased muscle mass might be associated with loss of bone mass. Significant differences in any of the SMI values in the normal bone mass group were not observed, unlike those in the low and very low bone mass groups. The proportion of participants with normal bone mass was only 8% in the present study. This indicates that the sample size for the normal bone mass group was too small to detect a significant difference between the low and very low bone mass groups.

Regarding muscle strength, grip strength and knee extension strength were not significantly different between the groups (Table 2), suggesting that muscle strength does not decrease when bone mass decreases. The age-related rate of reduction of muscle mass is different from that of muscle strength [24]. Moreover, resistance training with an elastic band increased knee extension strength but did not change SMI in older women [25]. Muscle strength was related to not only muscle mass, but also muscle quality [24]. These findings indicate that changes in SMI do not necessary reflect changes in muscle strength. Therefore, muscle strength was not changed in spite of the decrease in SMI. Participants in this study might be a relatively healthy population. Therefore, muscle strength was not reduced in spite of muscle mass loss by maintenance of muscle quality. A previous study reported that grip strength was not significantly different between older adults with osteoporosis and those without osteoporosis [19]. In addition, there were no associations between muscle strength, such as knee extension torque and grip strength, and bone mineral density in older women [26]. Our results are similar to the findings of these studies. Meanwhile, other studies reported that grip strength is associated with higher bone mineral density [22,27], which does not concur with the findings of the present study. A previous study reported that knee extension strength is associated with bone mineral density at the femoral neck site but not at the lumbar spine site in postmenopausal women [28]. These findings imply that muscle strength is associated with only certain bone sites. In the present study, calcaneus bone mass was measured. Therefore, all three groups had similar grip strength and knee extension strength when grouped by T-scores for the calcaneus site.

Regarding physical performance, there were no significant differences in either gait speed or TUG between the groups (Table 2). In previous studies, these physical performance indicators were also not correlated with bone mineral density [29,30] in older women. Meanwhile, a study reported that gait speed is associated with bone mineral density and found an association between grip strength and gait speed in postmenopausal women [27]. In the present study, muscle strength was similar for all groups. Muscle strength is associated with gait speed in older women [13]. Therefore, although the reason for the discrepancy between our results and those of a previous study [27] is not clear, our results seem to be appropriate because of the similar levels of muscle strength between the three groups.

In sarcopenic older women, the risk of osteoporosis was 12.9 times greater when compared to non-sarcopenic women [31]. The rate of osteoporosis was higher in the group of older women with sarcopenia than that in the group of older women without sarcopenia [32]. These findings suggest that low bone density and sarcopenia are related to each other. However, there was no association between bone mass and sarcopenia in the present study. The rate of sarcopenia has been reported to increase with advancing reduction of bone mineral density [32]. The prevalence of sarcopenia, which was determined according to criteria published by the European Working Group on Sarcopenia, was 22.1% in Japanese older women [33]. In the present study, only 4.2% of participants were evaluated with sarcopenia, and the rate was very low when compared to the previous study [33]. Participants recruited in the present study might be from a relatively healthy population, which may be the reason why there was no association between bone mass and sarcopenia in the present study.

In the present study, there are some limitations. First, this study used a cross-sectional design, and it was difficult to estimate causality between muscle mass and bone mass. Further longitudinal research is required to investigate causality. Second, the study recruited a small sample of older women with normal bone mass compared to sample sizes of low and very low bone mass groups. Therefore, there might be beta error when comparing between the normal group and the low bone mass and very low bone mass groups. Further research is required with a larger sample size to clarify the association of low bone mass with decreased skeletal muscle mass between the normal bone mass group and the low and very low groups. Third, participants recruited in this study were relatively healthy. It is unclear whether the findings apply to less healthy populations; similar research is necessary. Despite these limitations, this study is meaningful, as the findings suggest that muscle mass could be used as an indicator of bone mass loss in older women and that maintaining muscle mass could prevent low bone mass.

## 5. Conclusions

This study found that older women with low bone mass had decreased skeletal muscle mass. Appropriate nutrition intake is crucial to preserve muscle mass [34]. Physical exercise also positively influences muscle mass [35]. In addition, physical exercise induces secretion of myokines [36], which is associated with improved bone health. Therefore, health professionals should provide appropriate nutrition-based interventions for older women and encourage physical exercise, while monitoring postmenopausal women’s muscle mass regularly.

## Figures and Tables

**Table 1:** Participants’ characteristics.

Variable	Normal Bone Mass (*n* = 18)	Low Bone Mass (*n* = 112)	Very Low Bone Mass (*n* = 84)	F, χ^2^	*p*
Age (years)	72.6 ± 7.2	73.7 ± 5.0	75.5 ± 5.4	3.783	0.024
Height (cm)	151.6 ± 6.5	152.1 ± 4.5	150.9 ± 5.9	1.193	0.305
Weight (kg)	51.6 ± 6.2	52.4 ± 6.5	48.4 ± 6.9 ***	8.845	<0.001
BMI (kg/cm^2^)	22.4 ± 2.5	22.7 ± 2.7	21.2 ± 2.7 **	6.11	0.003
Diseases					
Hypertension (*n*, %)	9 (50%)	43 (38%)	25 (30%)	3.229	0.199
Hyperglycemia (*n*, %)	4 (22%)	27 (24%)	14 (17%)	1.617	0.446
Orthopedic disease (*n*, %)	2 (11%)	20 (18%)	20 (24%)	1.981	0.371
Diabetes mellitus (*n*, %)	2 (11%)	8 (7%)	3 (4%)	1.947	0.378
Cardiovascular disease (*n*, %)	0 (0%)	5 (4%)	8 (10%)	3.425	0.180
Pulmonary disease (*n*, %)	0 (0%)	2 (2%)	3 (4%)	1.141	0.565
Renal disease (*n*, %)	0 (0%)	2 (2%)	1 (1%)	0.402	0.817
Cancer (*n*, %)	0 (0%)	0 (0%)	1 (1%)	1.555	0.460
Others (*n*, %)	1 (6%)	17 (15%)	6 (7%)	3.745	0.154

Values of age, height, weight, and body mass index (BMI) are expressed as means ± standard deviation. Diseases were expressed by number and percentage of participants. Abbreviations: F, F-value; χ^2^, chi-square value. ** *p* < 0.01 and *** *p* < 0.01 vs. the low bone mass group.

**Table 2:** Comparison of skeletal muscle index (SMI), muscle strength, physical performance, and sarcopenia between the normal, low, and very low muscle mass groups.

Variable	Normal Bone Mass (*n* = 18)	Low Bone Mass (*n* = 112)	Very Low Bone Mass (*n* = 84)	F, χ^2^	*p*
Arm SMI (kg/cm^2^)	1.42 ± 0.20	1.41 ± 0.20	1.34 ± 0.19 *	3.525	0.031
Leg SMI (kg/cm^2^)	4.72 ± 0.43	4.69 ± 0.42	4.49 ± 0.39 *	4.681	0.010
Appendicular SMI (kg/cm^2^)	6.14 ± 0.59	6.10 ± 0.58	5.83 ± 0.54 **	4.931	0.008
Grip strength (kgf)	24.4 ± 4.2	24.0 ± 4.0	23.8 ± 4.0	0.063	0.939
Knee extension strength (kgf)	21.4 ± 5.5	21.3 ± 4.0	20.0 ± 5.0	0.780	0.460
Usual gait speed (m/s)	1.44 ± 0.18	1.49 ± 0.23	1.47 ± 0.27	0.560	0.572
Fastest gait speed (m/s)	1.85 ± 0.22	1.86 ± 0.27	1.82 ± 0.30	0.205	0.814
TUG (s)	5.55 ± 0.94	5.58 ± 0.96	5.83 ± 1.16	0.240	0.787
Sarcopenia (*n*, %)	0 (0%)	6 (5.4%)	3 (3.6%)	1.243	0.537

Values of skeletal muscle index (SMI), muscle strength, and physical performance are expressed as means ± standard deviation. Sarcopenia represents the number and percentage of participants who met criteria of sarcopenia as published by the Asian Working Group for Sarcopenia [17]. Abbreviations: TUG, timed up and go; F, F-value * *p* < 0.05 and ** *p* < 0.01 vs. the low bone mass group; χ^2^, chi-square value.
